# Access to land and nature as health determinants: a qualitative analysis exploring meaningful human-nature relationships among Indigenous youth in central Canada

**DOI:** 10.1186/s12889-024-20007-9

**Published:** 2024-09-18

**Authors:** Andrew R. Hatala, Darrien Morton, Cindy Deschenes, Kelley Bird-Naytowhow

**Affiliations:** 1 Department of Community Health Sciences, Max Rady College of Medicine, University of Manitoba, 750 Bannatyne Ave, Winnipeg, MB R3E 0W2 Canada; 2 Department of Community Health and Epidemiology, College of Medicine, University of Saskatchewan, 107 Wiggins Rd, Saskatoon, SK S7N 5E5 Canada

**Keywords:** Health Determinants, Indigenous Health, Youth, Land, Nature, Canada

## Abstract

**Background:**

Human relationships with and connections to nature and the “land” are a commonly accepted Social Determinant of Health. Greater knowledge about these relationships can inform public health policies and interventions focused on health equity among Indigenous populations. Two research questions were explored: (1) what are the experiences of meaningful human-nature relationships among Indigenous youth within central Canada; and (2) how do these relationships function as a determinant of health and wellness within their lives.

**Methods:**

Drawing from three community-based participatory research (CBPR) projects within two urban centers in Saskatchewan and Manitoba, the integrated qualitative findings presented here involved 92 interviews with 52 Indigenous youth that occurred over a period of nine years (2014–2023). Informed by “two-eyed seeing,” this analysis combined Indigenous Methodologies and a Constructivist Grounded Theory approach.

**Results:**

Our integrative analysis revealed three cross-cutting themes about meaningful human-nature relationships: (1) promoting cultural belonging and positive identity; (2) connecting to community and family; and (3) supporting spiritual health and relationships. The experiences of young people also emphasized barriers to land and nature access within their local environments.

**Discussion:**

Policies, practices, and interventions aimed at strengthening urban Indigenous young peoples’ relationships to and connections with nature and the land can have a positive impact on their health and wellness. Public Health systems and healthcare providers can learn about leveraging the health benefits of human-nature relationships at individual and community levels, and this is particularly vital for those working to advance health equity among Indigenous populations.

**Supplementary Information:**

The online version contains supplementary material available at 10.1186/s12889-024-20007-9.

## Introduction

Access to health care is a pressing concern [1]. Yet there is a growing recognition that access to care and quality of care are not enough to be healthy [2–4]. This view has led to an increased focus on the health behaviors, social and economic factors, and neighborhood conditions that considerably shape individual and community health and wellness [5–7]. These social determinants of health (SDH) are the contexts in which a person lives, develops, and works, including the local and global political and economic fluctuations of power and resources [7].

Human-nature connections and relationships are a commonly accepted SDH [6–9]. Nature, broadly defined, includes any outdoor space with vegetation such as large municipal parks, informal neighbourhood trails, community gardens, greened schoolyards, and larger bodies of natural parks, rivers, lakes, oceans, or assorted green spaces outside urban centers [10].

Contemporary scientific evidence affirms the connection between nature and human health, and actionable understandings of these links are advancing in diverse disciplines [11–18]. For example, nature exposure can impact health through boosted immune response, improved air quality, reduced urban heat island effect, lowered stress, enhanced mental health and child development, increased physical activity, improved affect and cognition, and the development of social connections [19–28]. Nature exposure has also been proposed as a tool to reduce entrenched geographic and socio-economic health inequities [29–32]. Many community or individual level health interventions in these areas, however, do not involve Indigenous populations or adequately draw on Indigenous knowledge systems or cultural expertise.

Conceptions of and relationships to nature or the “land” within Indigenous health literature makes vital contributions to the growing evidence on human-nature interactions and their health impacts, especially critiques of colonial and secular views of natural environments emphasizing separateness and a capitalist commodification of the land [33–42]. Additional calls for broader understandings of the SDH for Indigenous Peoples take into account the historical and contemporary agendas of settler colonialism and systemic racism that impede access to land and nature—thereby diminishing health and wellness trajectories for diverse Indigenous populations [6, 31, 44]. Conversely, several studies now illustrate how individual and community level relationships with the land and nature function as determinants of health by improving housing security, social cohesion, spiritual or cultural identity, and access to traditional foods and medicines [37–44]. 

Given the importance of access to nature and land as vital health promoting assets for many peoples’ lives, understanding the experiences of meaningful human-nature relationships and interactions—as well as the factors that permit or deny access to nature and the land—should be a priority for public health interventions and system reforms.

Building effective knowledge about human-nature relationships, including what access means for diverse populations, can inform policies and programs focused on health equity among Indigenous populations. This knowledge can also assist health care professionals and systems better understand and work with this important health promoting relationship. Indeed, there is demand from practitioners and decision makers to incorporate emerging evidence regarding the health effects of nature experiences into health care service assessments and policy [8, 10]. Yet health promoting aspects of nature exposures and relationships do not receive as much attention by health care providers or public health systems as other social concerns [8]. Most research in this area also employs quantitative epidemiological-type outcome or correlational metrics; in-depth community-engaged, qualitative evidence on the experiences of young people and factors that permit or deny access to nature and land remains limited.

To address these issues and advance this knowledge base, two primary research questions were explored: (1) what are the experiences of meaningful human-nature relationships among Indigenous youth within urban contexts in central Canada; and (2) how do these relationships function as a determinant of health and wellness within their lives.

## Methods

### Study design and setting

This study draws from three Community-Based Participatory Research (CBPR) projects exploring Indigenous youth health and wellness within two urban centers in Saskatchewan and Manitoba, central Canada [38, 45–48]. Identifying the need to build on existing community strengths, these projects were carried out in 2014–16, 2017–19, and 2019–2023 involving youth-serving organizations, several years of relationship building, and engagement with youth, parents, and local Elders.

All three of these projects followed a “Two-Eyed Seeing” approach whereby Indigenous and Western methodologies or frameworks worked alongside one another [45, 49]. Applied to our research team consisting of Indigenous and non-Indigenous researchers and youth collaborators, this approach created spaces for open discussions regarding the roles and responsibilities of both ways-of-seeing and ways-of-doing, enhancing our overall methods. The Consolidated Criteria for Reporting Qualitative Research (COREQ) was also applied throughout knowledge dissemination [50], and is attached as an appendix for reference.

### Data generation and analysis

All three projects utilized Indigenous Methodologies [51] alongside a modified Constructivist Grounded Theory (CGT) approach [52] for data collection. The CGT approach was “modified” to ensure decisions about methods and how they were employed in data collection and analysis occurred through Indigenous ontological and epistemological perspectives prioritizing relationality and a strength-based orientation [51]. 

In total, the integrated findings presented here involved 92 interviews with 52 Indigenous youth (Plains Cree, Métis, and Anishnabe) that occurred over a period of 9 years (2014–2023) (see Table 1 for more details). The interview guide used for all three CBPR studies is included in Appendix 1. Aspects of human-nature relationships were not an initial focus of the CBPR projects, but rather emerged as a consistent theme throughout interviews and individual analysis of each project over the decade. Further integrative analysis explored here then focused more directly on the human-nature theme and its role as a health promoting relationship in young Indigenous Peoples’ lives.

**Table 1:** Data collection procedures for CBPR projects 2014–15 and 2017–18 and 2019–2023

Data collection procedures	CBPR Project 1 (2014–2015)	CBPR Project 2 (2017–2018)	CBPR Project 3 (2019–2023)
Sampling Methods	Combination of purposeful andsnowball sampling was used to recruit youth self-identifying as Indigenous through partnering organizations.	Convenience and purposeful sampling was used to recruit youth self-identifying as Indigenous through partnering organization and previous community relationshipss.	Convenience and purposeful sampling was used to recruit youth self-identifying as Indigenous through partnering organizations and previous community relationships.
Sample Population Demographics	28 participants; ages 15–25 years (16 females, 12 males), and self-identifying as Nêhiyaw Plains Cree (*n* = 21) and Métis (*n* = 7)	8 participants; ages 18–24 (6 females, 2 males), and self- identifying as Nêhiyaw Plains Cree (*n* = 6) and Métis (*n* = 2)	16 participants; ages 18–26 (7 females, 9 males), and self-identifying as Nêhiyaw Plains Cree (*n* = 6) and Métis (*n* = 4) and Anishnabe (6)
Conversational Interviews	After consent was granted and discussed, interviews with youth were audio recorded with permission and later transcribed verbatim. As recommended by our Elders, interviews began by creating “safe spaces” where Nêhiyaw, Métis and Anishnabe cultural lifeways or protocols were followed, including smudging with sacred medicines if desired and offering non-commercial tobacco to youth to respect the sacredness of their stories, experiences, and knowledge. Additionally, aspects of how we employed Indigenous Methodologies in this research and “modified” our Constructivist Grounded Theory framework, have been detailed in previous publications from our team.^45^ Interviews were conducted with at least two university researchers (1st and 2nd authors) and/or Indigenous youth research assistants to ensure meaningful conversations, intergenerational mentorship, and diverse perspectives. Guidance from Indigenous team leads was also followed throughout (3rd and 4th authors). We also allowed time and space for participants to build relationships with researchers outside interview settings by engaging in ceremonial activities together as well as participating in other community feasts and gatherings throughout the duration of the research.		
Total Interview Transcripts	*N* = 38, including 10 follow-up interviews	*N* = 16, including 8 follow up interviews	*N* = 38, including 16 follow up interviews
Relational ethics and accountability	Following Indigenous Methodologies, relational ethics and accountability were strengthened by honoring relationships among youth and the landscapes we were working with and on throughout the three projects.^50^ This included, but was not limited to: collective decision-making processes reflective of urban Indigenous sovereignty; intergenerational mentorship; building cultural, personal, and professional capacities of youth as co-collaborators; ensuring appropriate introductions and preparations were performed with all collaborators to create safety; and extending relationships beyond the three projects to involve ongoing research partnerships, ceremonial and community engagement, and sustaining friendships.		
Data Rigour and trustworthiness	To maintain rigour or trustworthiness in the analysis, multiple steps were taken to ensure credibility, transferability, dependability, and confirmability of the data. These aspects helped to promote *critical reflexivity*, essential in helping our team deepen our understanding of our own biases and where/if those affected any decisions made throughout the different projects.^45^ Regular team meetings supported triangulation throughout the projects and during secondary analysis. Multiple coders (1st and 2nd authors) and comparisons across applications were employed throughout analysis that included conversations with the 3rd and 4th authors. Regular *memo-taking* and field notes from interviews, team meetings and gatherings added a further layer of richness and understanding to the data and stories shared.^51–52^*Transferability* here was not about being able to exactly replicate the results or process elsewhere, but rather to illustrate that, under similar contexts, these findings could act as a starting point for the application of these findings to health promoting interventions across Canada or abroad.		

This analysis involved an integration through merging process whereby the qualitative data from the three CBPR projects were brought together for overall re-analysis and comparative thematic synthesis [53, 54]. Utilizing CGT, the interpretation of the collated data followed an inductive approach with open and axial coding strategies [52]. Initial themes were coded using Dedoose software Version 9.0 (2023) by the first author and checked for consistency by the others. Two-Eyed Seeing in analysis ensured that both Western and Indigenous ways-of-seeing and relating with the data were employed [49, 50]. This form of secondary analysis of qualitative data is common in health research and established guidelines were adhered to throughout [53, 54]. Due to thematic triangulation in analysis across the three projects, a robust and nuanced understanding of the research questions emerged.

### Ethics approval

Ethics approvals were received from the University of Saskatchewan and the University of Manitoba for the CBPR projects, including secondary analyses of data. Additional ethical guidance from local Elders, youth, and youth-serving organizations were also obtained.

## Results

The integrative analysis highlights several pathways of how access to land and nature function as determinants of Indigenous youth health and wellness, revealing three cross-cutting themes: (1) promoting cultural belonging and positive identity; (2) connecting to community and family; and (3) supporting spiritual health and relationships.

For youth in this research, nature experiences and land connections occurred in diverse ways, including: exploring parks and trails within urban centers; attending powwows and going on hunting trips out of the city; attending Sundance ceremonies for extended periods over the summer months; and joining day trips out of the city for sweat lodge ceremonies or harvesting sacred medicines. The findings presented here outline similarities across these diverse nature and land-based experiences and do not focus on the variations that may exist across them.

### Promoting cultural belonging and positive identity

Central to our research were youth expressions regarding how connecting with nature and “being on the land” promoted wellness through cultural identity and belonging. Juxtaposed against inundating experiences of “feeling displaced” as Indigenous young people growing up within urban Canadian contexts, being on the land and connecting with nature helped young people “feel like they belonged”; it helped secure a meaningful place and an Indigenous identity rooted in positive value and history (see Table 2).

**Table 2:** Selected participant quotes about promoting cultural belonging and positive identity

• “When you’re out there with other brown bodies in nature like that, where we’ve been gathering for hundreds of years, you don’t have to like worry as much about judgements or what others think. It’s safer out there and you can just be you.” – P09
• “I felt better about myself. I was like, feeling like I belong. Like, there’s a place for me in the ceremony and ceremonial places [on the land]. I can be confident about myself there.” – P12
• “There’s a point you realize you don’t need to go to a counselor and get prescribed pills, but going out on the land in nature and in ceremony, connecting with the culture and identity, yeah like that can boost our mental health too.” – P01
• “Like, how I think it [hunting] like helped me find this place of belonging, I think and I say that because it’s like that very displaced feeling of being an Indigenous youth growing up in the city with no connections or like little teachings.” – P03
• “Once we went to the hunt it definitely brought like a revitalization of teachings to me, I guess, as an Indigenous person.” – P05
• “For myself. Personally, I think that hunt brought lots of teachings to us as young people, like about who we are as Indigenous people.” – P06
• “I was grateful to have the opportunity to have someone take us out onto the land and teach those ways. I belonged out there you know, as an Indigenous person, its different from being in the bush from being in the city. I was meant to be out there like that and I could feel that.” – P10
• “I’m realizing that my relationship between myself and the land, I’m still getting into, like, I’m still getting to know or reintroducing myself, if that makes sense. So, just having the opportunity to learn about land-based teachings or ceremonial teachings or whatever, it’s like learning more about myself in a way too.” – P11
• “The jurisdiction will always come from the land. So the jurisdiction, meaning, your knowledge, your knowledge, base, your knowledge of self, knowledge of others, and knowledge of community knowledge of conducting yourself, will always come from the land. So those are the connections we have with land, the closer connection we have with knowledge, with self, with community, with relationships. So, your jurisdiction as a person, who you are, will always come from the land. – P05
• “Well, I’ve come to really understand that my sacred story really goes back to my ceremonial days where like I would go to the sweat lodges, Sundance, round dance, powwows, or even the things that you do before like working out on the land, cutting wood, going and get the rocks, setting up the arbour. Doing that helped me, that really gave me like that identity of being Indigenous and the traditional values that it gave me like the teachings, the healing process of ceremony, going on that healing journey; that’s what really did help me.” – P20
• “Our culture has that connection to the land, that connection to creation and Mother Earth, the moon, the sunset, like all of those things, and the language, they all have like I don’t know, like this spiritual connection to the land who we are as a people.” – P19
• “Through ceremony I fell in love with the culture and learning more about being First Nations and having that connection with the land, and I felt like I belonged.” – P01
• “You know, just that connection with the land and everything, really helped me.” – P03

Being with nature on the land also exposed youth to cultural teachings about how to live a good life, how to take care of one’s self, others, and the future generations—“the land is our teacher” as one youth remarked. This connection with “the teachings” on the land buoyed youth from various traumatic life experiences—from micro aggressions, to systemic racism, from loss of loved ones and gang violence, or chronic poverty and injustices—while promoting a context for healing, resilience, and mental health support. Fundamentally, many of these teachings only emerge while being in relation with and directly out on the land, and cannot be accessed the same way in class rooms or smaller urban contexts.

This positive sense of Indigenous identity and belonging, derived from various nature experiences and cultural encounters with the land, was a protection against harmful environments threatening youth health and wellness outcomes. Repeatedly, youth with regular access to land and nature conveyed a positive sense of self and commitment to walking a “traditional path” rooted in positive cultural values, constructive resilience, and healing.

### Connecting to community and family

Youth also spoke extensively over the years about how nature and land exposures were rich with wellness-promoting connections to community and family. Ceremonies on the land, and especially multi-day gatherings like Sundance and hunting trips, required a large network of individuals and families to set up and organize, and were often described as “multiple hands-on deck” occasions. Working as a village or community was often required to ensure these on-the-land gatherings and camps could occur “in a good way” (See Table 3).

**Table 3:** Themes with selected participant quotes regarding connecting to community and family

• “In addition to the connections I made to the land there, grounding myself, you know, crying and sweating it out there, the benefits from visiting Sundance were the connections I made with the people, friendships and relationships that can very well last until I die. That’s the truth right there, that lasting respect and love.” – P12
• “As humans it’s not very common that you can make those connections with people in your everyday life. It’s actually quite uncommon, especially these days. And so being able to be out there, you know, on the land, you know, we’re sitting there visiting with people and those Elders in a way that you know, you just don’t get as often and as common anymore.” - P14
• “This [hunting and Sundance ceremony] would need multiple hands-on deck to get this done, yeah.” – P04
• “And then as for harvesting, you have multiple community members helping you out with that. And everybody in the community was able to come over and help me out with and that makes job quite easier.” – P27
• “I wanted to enter into the lodge to, I don’t know if reset is the right word, but just kind of reground myself into the new territory that I visit into this new land that I had never been in before. It made me feel connected to these new people that I was meeting and it made me feel connected to the place.” – P02
• “During that Sundance I was a visitor, on that, this new territory…And so I really wanted to have my own connection to it. And anytime you visit anywhere, I feel like that’s important to just reground yourself in a new territory, because it’s a transition for your body, it’s a transition for the space to have you inhabited there as well, and to have, you know, my energy walking through that space, not only for that land, but also for the people also walking around my same space.” – P03
• “In ceremony, you become relatives.” - P08

It was often through these experiences, of helping set up camp and prepare for ceremony on the land, that valuable connections with members of the community were built. “In ceremony, you become relatives,” one young person explained. Here nature and land-based experiences were seen to foster connections between youth, older adults, community leaders, and Elders that also uniquely reinforced aspects of wellness, belonging, and identity when compared to other educational programs or health-related opportunities.

Access to nature and on the land experiences, then, were not solely for youth as individuals, as important as that can be, but also about connecting youth to positive role models and supportive adults, community leaders, and Elders, that strengthen their capabilities at living a healthy or “good” life. Through these connections, youth received important life lessons, cultural stories and were supported in learning to think differently, critically, and independently about themselves and the world around them. It was centrally the relational context of being on and with the land that allowed such community and family connections to flourish.

### Supporting spiritual health and relationships

Another key finding from this research was how nature and land exposures support spiritual health and relationships, often through personal or collective ceremony in both rural or urban settings. Spiritual health typically involves a ‘way of being’ and deeply felt human connections to or relationships with aspects of the self and others, a sense of nature, and a sense of whatever one considers to be ‘transcendent’ [55]. It involves a personal relationship with the seen and unseen. Connecting with nature for these youth was thus also about building deeper forms of transcendent relationships with spiritual beings and the Creator. Beyond the community, these spiritual relationships were further described as supportive in one’s life—kinship with trees and the spiritual values associated with these relationships, such as humility, reciprocity, and exchange, became central to youth wellness. Spiritual practices and relationships on the land helped young people reflect on difficult experiences, reframe their outlook in a more positive manner, develop meaning through stressful or difficult situations, and rely upon more optimal behavioural or coping strategies (See Table 4).

**Table 4:** Selected participant quotes regarding supporting spiritual health and relationships

• “Going into the bush [on the land], one thing that I’ve learned is that there’s a certain relationship there, where you’re walking into this community of relatives like those trees, or our relatives, the medicine there, and the animals who live in that community and respecting that space going into there. We put down our tobacco for that space, to respect our relatives in the trees.” – P04
• “I’ve really learned that you’ve got to kind of make your own ceremony with whatever you’re given in that time. Because you’re not always going to have a Powwow to run to [when difficulties emerge]. You’re not always going to have what you need available to you… So, for me, it’s even just going to sit down somewhere outside in a park of by a river or whatever to clear my mind that can help me out.” – P03
• “Family members who are sick, even like it [ceremony on the land] just like it helps people in the community.” - P04
• “I think about the importance of like ceremony and being on the land and like how it helps heal some of these families who deal with like addictions and missing and murdered [referring to missing and murdered Indigenous women].” - P06
• “Yeah, there’s a deeper meaning to “land back”. And actually, having that spirituality aspect to it too. I didn’t know how important it is to practice that spirituality that exchange between life and death, like it’s very important for an Indigenous person to know about.” – P20
• “Spiritually we prepped for that hunt by smudging the weapons and putting tobacco down, because it’s about a relationship with the animals, with nature…spiritually we prep for it because we are in a relationship, it was like from relative to relative we went into that hunt asking for food from our relatives.” - P02
• “It’s basically a ceremony what we’re doing is we’re going out on the land for food, and it’s all a preparation in doing this hunt because we’re taking a life and that’s something we’re asking Creator for respectfully and we’re giving back by offering that tobacco.” – P08
• “We talked about protecting the land, protecting the water, it’s for the babies, every time. You know, we fight for rights, we fight for Indigenous rights, we fight for our relatives, it’s for the babies.” – P02
• “But I think what I’ve realized is that as Indigenous people we’re always researching and we always have been researching before colonization, like our people knew our land like…like the back of their hand. And doing these ceremonies and coming here on the land gives us a deeper sense of that way of doing things, of living life and all.” - P16
• “When you become an adult, you have these new relationships and responsibilities, and bonds. Maybe providing for your own home or maybe this new role or responsibility or relationship with the land and knowing the plants. There are different roles of being an adult.” – P10
• “If you’re going to this sweat or going to Sundance, you can go there on that land to pray and everything. And so if you’re not doing good, either mentally or even physically, you can go there and you can, you know, pray.” – P09

Although ceremony among many Indigenous communities can involve larger events and gatherings outside of the city (e.g., Sweat Lodges or Sundance), youth also expressed a more personal ceremony in urban parks or trails involving prayer, smudging, or fasting. Larger ceremonies or sacred gatherings on the land may not always be possible for many Indigenous youth in the city. As such, these subtle forms of personal connection with nature and the land in urban contexts can be supportive of mental wellness, identity, and relationality [38, 48]. Here, “making the best of any situation” with “whatever you’re given in that time” becomes an important practice of constructive resilience for these young people within urban contexts.

While human-nature connections within cities were possible and are important, all youth stressed how deeper spiritual connections with the Creator occur more easily outside urban contexts being completely embraced by nature and on the land. Having access to experiences on the land outside the city walls was therefore central to healing and positive growth for all the young people in this research.

## Discussion

It is generally accepted that human health improves with access to and meaningful connections with nature [10–17, 28]. This is especially true for Indigenous populations where nature is more than an aspect of the physical environment; but includes important kinship relationships, sources of identity and belonging, and ceremonial encounters [40]. Indeed, ceremonial land-based practices explored in this research strengthened young peoples’ cultural connections, positive identity formations, and holistic health-promoting lifeways, such as traditional knowledge, food preparations, and medicine gathering. Connections to land and nature are not, therefore, an objective outcome that is easily measurable, like physical activity or water quality. Instead, what youth described here is that land and nature are complex relational concepts spanning across the physical, social, emotional, mental, and spiritual dimensions of health and wellness—they are more than a social or emotional support system, a physical landscape, or the place of material resources for survival [56–59]. This interconnected and sacred connection to nature and the land has also been identified as a *relational determinant of Indigenous health* (RDH), moving beyond a focus solely on the “social” while centering on existing strengths at community and individual levels [36]. 

Drawing attention to the land and nature as a primary determinant of Indigenous health highlights the need to better understand and promote the factors that support access and meaningful human relationships to the land and nature, including: increased self-determination; ownership and stewardship of lands; and implementation of Indigenous-led initiatives that facilitate such connections [9, 33–43, 60]. This importance of jurisdiction and access to lands was also reaffirmed when the United Nations declared it an essential right of Indigenous Peoples to maintain and strengthen their distinctive spiritual relationship with their traditional lands, territories, waters and coastal seas [61]. Similarly, The Truth and Reconciliation Commission of Canada (TRC) called for full health care rights for Indigenous Peoples, self-determination including access to traditional therapies and land-based healing practices, as well as anti-racist decolonization of the health sector [44]. Indigenous scholars and leaders have also stressed that Indigenous ownership and protection of land is more pressing than ever before due to the rapid and unprecedented rate of loss of land, urbanization, resource mining, and climate fluctuations and disasters [57]. 

To assert self-determination over land, identity and wellness, Indigenous communities are building strategies to reclaim and reconnect with their territories. Across Canada and globally, Indigenous communities are using land-based forms of sustenance and healing to promote individual and community health and wellness, in both urban and rural areas [57, 62–69]. These forms of healing challenge medical colonialism and Indigenous racism that continue to constrain Indigenous Peoples’ equitable access to safe and effective health care, as well as spaces of nature or land in colonized countries like Canada [67]. The notion of *environmental repossession* seeks to describe these activities and explore how they develop in response to the unique needs of various Indigenous communities [68]. 

Despite acceptance of nature as an RDH or SDH, the abundant evidence linking nature and health, and the calls for health providers to discuss nature with patients from public health and medical professional organizations, nature is generally missing from the rapidly growing pool of structural interventions [8]. Public health systems and healthcare providers could therefore more seriously consider how to leverage the health benefits of human-nature relationships at individual and community levels, and this is particularly vital for those working to advance health equity among Indigenous populations [29–31]. Indeed, due to long histories of settler colonialism, dispossession, oppression, and contemporary forms of racism, certain aspects of urban environments, like city parks or walking trails, are not always safe spaces for Indigenous Peoples [9, 38, 48]. A greater recognition of the relationship between nature exposure and health is also therefore likely to highlight income-related inequalities and provide one of many possible pathways to reduce them. Removing social and physical barriers to nature exposures and relationships is thus not only an issue of health care access, but also of environmental justice [70–73]. 

Moving forward, public health teams and health care providers could better understand and emphasize human-nature connections among diverse communities they work with and better appreciate their health promoting potential. Since accessing nature will look different in urban, suburban, and rural locations, care providers should individualize nature exposure recommendations for each patient or population, and openly explore barriers and facilitators. Examples of this are emerging from the ParkRx movement that involve providers prescribing time in nature, including at times office organized nature visits [74]. One Randomized Control Trial (RCT) study found that among low-income minority families, clinician-recommended park visits and office-led group visits to city parks, reduced stress among parents and improved resilience among children [75]. Community or public health workers who have knowledge about local neighborhood conditions, as well as the spatial or social access issues of a population, would be adept at addressing barriers to behavior change in culturally meaningful ways. Although important, much of this work still fails to include Indigenous populations and cultural or spiritual components in meaningful ways. For those who work and walk with Indigenous practitioners and patients, it would therefore be important to recall that a deeper cultural and spiritual understanding of nature and the land differentiates these initiatives from generalized “outdoor”, “adventure”, “wilderness” or “nature-based” health programs or therapies informed from Western conceptualizations and values laden with separateness or “stewardship over” rather than *interdependence with* the natural world [39–42, 62–67]. 

Health systems, in partnership with external stake-holders and city planners, could also invest in new, safe, and accessible green space as well as renovate and maintain existing green space in the neighborhoods where their patients live. Vacant lot greening, a simple, scalable, and low-cost urban nature project, is an example of an evidence-based intervention that health systems can invest in to promote health within their jurisdictions. In one citywide RCT, for example, vacant lots were randomly assigned to either a greening intervention involving cleaning previously blighted spaces and adding new grass, trees, and a low wooden fence; a trash cleanup-only intervention; or no intervention [76, 77]. Crime rates went down, and people living near greened lots reported feeling less depressed, feeling safer, and having more social interaction with neighbors after greening. This low-cost intervention provided new green spaces, thus increasing nature access and exposures in cities, while also providing clean and well-maintained spaces that translated into social connectedness [77, 78]. Building on this type of work, public health offices can also grow capacity for community or Indigenous-led greening efforts by referring patients to organizations whose mission is to increase nature access through activities such as tree planting, park cleanups, and community gardening [79]. In this way, efforts within primary care offices would support both individual patients and community-led advocacy for increased access to clean and safe green spaces in their jurisdictions, both rural and urban.

### Limitations

Participants in this study were from communities in central Saskatchewan and Manitoba and are therefore not representative of other Indigenous youth across Canada. Experiences of youth in the arctic or coastal communities may bring important nuances to this work. Connections to land and nature are also complex, and there are different understandings between individuals, families, communities, and Nations related to how land is valued, used, and accessed [33, 79]. Similarities and differences between Indigenous understandings of human-nature relationships and those from other populations or ethnic groups were not explored here and would also be important moving forward [80]. Determining more clearly which aspects of land and nature connections are most relevant to health, mental health, and wellness and why was also not the focus of the current study. Although several evaluations have demonstrated that land-based programs increase health and wellness in youth [9, 81–83], there is still a need for more research around the conceptualization, design, delivery, and evaluation of these initiatives. Finally, youth in this project did not openly voice negative or neutral relationships with or experiences regarding access to nature and its health impacts. Future work could explore this area more directly, especially in relation to environmental devastation due to resource extraction or climate change.

## Conclusion

Too many Indigenous Peoples in Canada, including First Nations, Inuit, and Métis, face alarming health inequities, subpar access to health care, and culturally discontinuous services [6, 69]. This research suggests that policies, practices, and interventions aimed at strengthening urban Indigenous young peoples’ relationships to and connections with land can have a positive impact on their health and wellness, and can be one pathway to begin addressing existing inequities. Given the importance of human-nature relationships for health and wellness, connections to nature and the “land” should be seen as part of the broader healthcare ecosystem. Finding creative ways to overcome barriers to access and foster meaningful human-nature connections can thus continue to strengthen as an important health promotion strategy among Indigenous young people. More generally, imagining real change within healthcare and public health systems must therefore include equitable access to nature and greenspace within both urban and rural landscapes. Part of the “big and radical changes” that are needed for healthcare reforms therefore [1], could involve looking beyond the white gowns and grey bricks of hospital and clinic walls, to the green trees and blue lakes in the surrounding care ecologies.

## Electronic supplementary material

Below is the link to the electronic supplementary material.

Supplementary Material 1

Supplementary Material 2

## Data Availability

The datasets generated and/or analysed during the current study are not publicly available because of privacy concerns; participants are potentially identifiable due to the small sample and overall population size, and because of the qualitative nature of much of the data. The datasets are potentially available from the corresponding author on reasonable request.
