# Supplementary material for: Access to land and nature as health determinants: a qualitative analysis exploring meaningful human-nature relationships among Indigenous youth in central Canada
Source: BMC Public Health. 2024 Sep 18;24:2540. doi: 10.1186/s12889-024-20007-9 (PMC11411752; doi:10.1186/s12889-024-20007-9)
Supplement: Supplementary file 1 — Supplementary Material 1 [file 12889_2024_20007_MOESM1_ESM.docx]

**Appendix 1: Youth Health and Wellness Interview Guide: Questions used in three CBPR Projects (2014-2015), (2017-2018), and (2019-2023).**

**General questions:**

1. Can you share with me ‘Who’ you are and ‘Where’ do you identify yourself as from?
2. What do I need to know to grow up well here?
3. How do you describe people who grow up well here despite the many problems they may face?
4. What kinds of things are most challenging for you growing up here?
5. What do you do when you face difficulties in your life?
6. What does being healthy mean to you and others in your family and community?
7. What do you do, and others you know do, to keep healthy, mentally, physically, emotionally, spiritually?
8. What kinds of things might make you feel unsafe? What do you or others you know do when they are not feeling safe?
9. What kinds of places or people can you visit when you are not feeling safe?
10. What protocols/precautions does one take when connecting with people, places or things?
11. What would you say are some of the strengths of your community?

**Contexts: Connection to Culture**

1. What cultural background would you say has most influenced you? What are some other cultural influences? How would you say your culture has shaped who you are?
2. Would you explain a little what it means to be ___________? (nationality)
3. Is your cultural identity important for you? Can you explain why this might be the case?
4. Are there cultural events that you attend? Do you enjoy participating in cultural events? Does your family support these events? Do your friends attend? What might these events be important?
5. What kinds of people do you look up to? How have these people impacted your life?
6. Have you ever felt discriminated because of your cultural background? How did this make you feel? What do you want to do about this? Do you talk with people about how this makes you feel?
7. Have you ever felt encouraged and supported because of your cultural background? How did this make you feel?
8. Would you say cultural traditions are important for you? Can you explain why you think these might be important? Are they important for everyone or only people from your background?
9. Are there any risks to participating in cultural activities?
10. Are there any benefits to participating in cultural activities?

**Contexts: Connection to spirituality**

- 1. How do you understand spirituality? (Same as religion? Prayer? Individual or family?)
  2. How do you practice your spirituality? (go to church, pray, serve others, smudge, feasts, holy days, religious laws, ceremony, sweats)
  3. Can you explain a little about how spirituality is used in difficult times? Why, how? What aspects of spirituality might help the most?
  4. Do you think spirituality has helped you during difficult times (Have you ever relied on prayer to make you feel better, religious or spiritual ceremony, etc.?)
  5. What do you think it means to “live a good life”?
  6. Would you say spirituality is an important part of your life? Your family life? Your community life?

**Notes:** Since these CBPR projects all utilized a qualitative framework and a more exploratory methodology and paradigm, the research questions were not meant to be tested in a positivist manner. As can be seen in the interview guide, aspects of human-nature relationships were not an initial focus of the CBPR projects. Instead, this topic emerged as a consistent theme throughout interviews and individual analysis of each project over the years. Further integrative analysis then focused more directly on the human-nature theme and its role as a health promoting resource in young Indigenous Peoples’ lives.
