# Supplementary figures and images for: Access to land and nature as health determinants: a qualitative analysis exploring meaningful human-nature relationships among Indigenous youth in central Canada
Source: BMC Public Health. 2024 Sep 18;24:2540. doi: 10.1186/s12889-024-20007-9 (PMC11411752; doi:10.1186/s12889-024-20007-9)

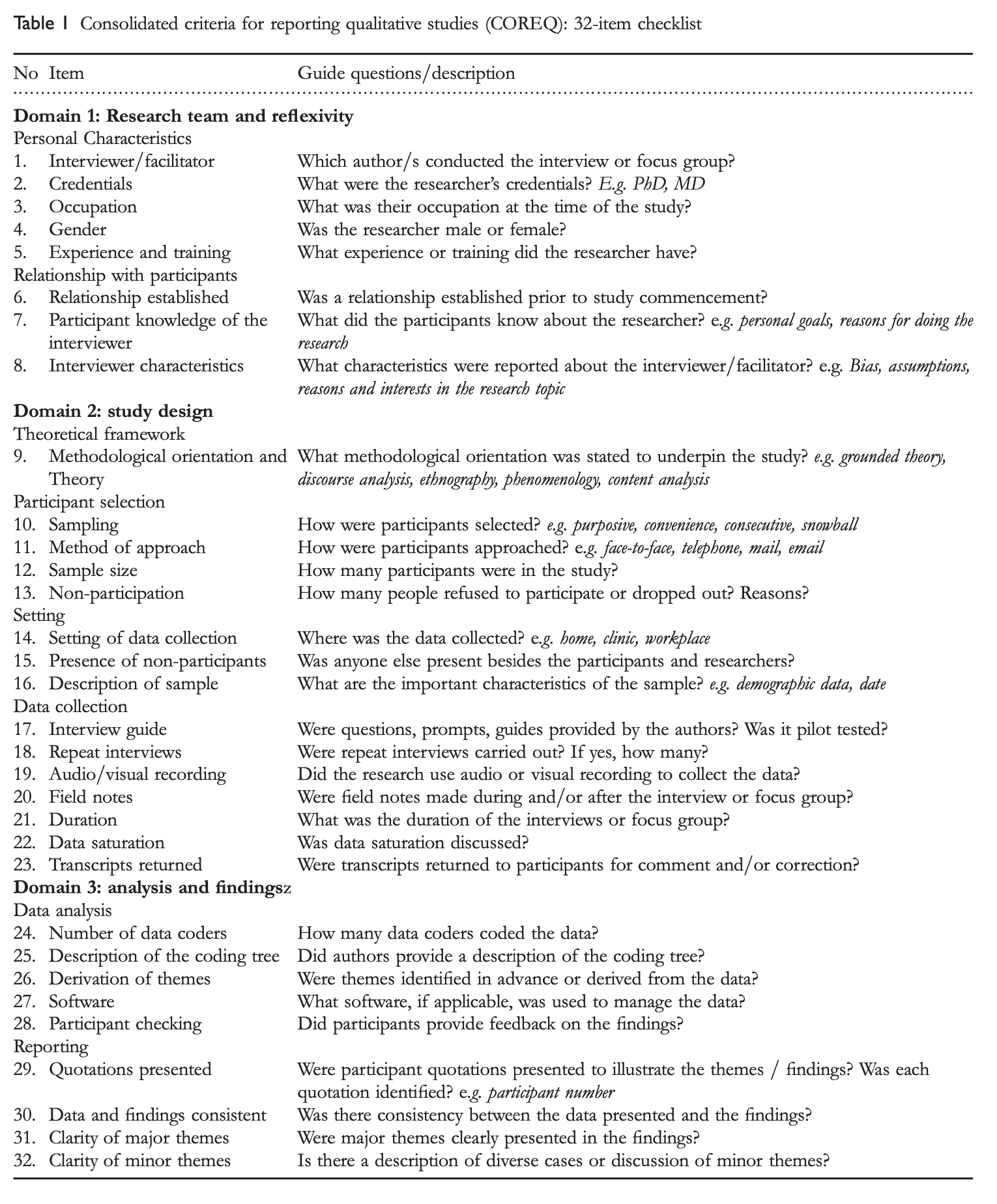

Supplement: Supplementary file 2 — Supplementary Material 2 [file 12889_2024_20007_MOESM2_ESM.docx]
